# The relevance of the Collaborative Online International Learning methodology in global training in Orofacial Motricity

**DOI:** 10.1590/2317-1782/e20250117en

**Published:** 2026-01-26

**Authors:** Lucas Ferreira, Felipe Henríquez, Paola Leguízamo, Maria Luiza Lopes Timóteo de Lima, Hilton Justino da Silva

**Affiliations:** 1 Programa de Pós-graduação em Saúde da Comunicação Humana – PPGSCH, Universidade Federal de Pernambuco – UFPE - Recife (PE), Brasil.; 2 Departamento de Salud, Universidad de Los Lagos - Osorno, Chile.; 3 Programa de Fonoaudiología, Escuela Colombiana de Rehabilitación – ECR – Bogotá D.C., Colombia.; 4 Programa de Pós-graduação em Saúde da Comunicação Humana, Departamento de Fonoaudiologia, Universidade Federal de Pernambuco – UFPE - Recife (PE), Brasil.

## Dear Editors-in-Chief of the CoDAS Journal,

The internationalization of teaching and research in Speech-Language Pathology has become an essential strategy for advancing the field, particularly in the area of Orofacial Motricity (OM), understood as the branch of Speech-Language Pathology dedicated to the study, research, prevention, assessment, diagnosis, development, habilitation, improvement, and rehabilitation of the structural and functional aspects of the orofacial and cervical regions^(1)^.

Access to different theoretical, methodological, and clinical approaches expands the possibilities for training and innovation. In this context, the Collaborative Online International Learning (COIL) methodology emerges as a pedagogical tool capable of connecting students, professors, and researchers worldwide, promoting a collaborative and intercultural learning environment^(2)^. In the field of OM, this methodology represents an innovative and relevant strategy for the comparative study of orofacial parameters among populations from different countries, fostering critical analysis of assessment and intervention methods and enabling the identification of cultural, clinical, and epidemiological similarities and differences related to orofacial disorders.

The COIL methodology stands out as an innovative approach that uses online technologies to enable global learning and the simultaneous exchange of knowledge, cultures, and ways of thinking within the academic sphere^(3)^. Through this virtual interaction between institutions from different countries, joint knowledge construction occurs without the need for physical mobility. Furthermore, COIL integrates the socioconstructivist educational approach, which posits that scientific knowledge is not a neutral set of data independent of cultural practices, but rather a social construction that emerges within the context of societies^(4)^, emphasizing learning through social interaction, an essential aspect for the development of intercultural competence^(5)^.

This methodology connects educators from different regions to develop a shared online program^(5)^. This process includes virtual group activities in which students collaborate to co-create learning. For example, a professor from university A may establish a partnership with a professor from an international university B to design a joint course or class, in which cultural exchange is also emphasized. In this context, two institutions, two professors, and two groups of students participate actively, which may involve more than one language, different perspectives, and diverse academic topics, all in a collaborative manner, with tasks carried out in a virtual environment that promotes student cooperation and interdisciplinarity.

Through this type of collaborative project, students can explore various aspects of OM, such as mouth breathing, mastication disorders, dysfunctional swallowing, sleep disorders, or speech alterations of musculoskeletal origin, among other topics. This experience encourages discussion on the development of valid assessment methods and clinical tools adapted to each context, promoting a practice grounded in equity and diversity.

In this way, the pursuit of a globalized OM can be achieved when teaching and knowledge production among different higher education institutions follow a more aligned approach, reducing discrepancies related to nomenclature, assessment processes, and therapeutic strategies for orofacial myofunctional disorders. This methodology can serve as a facilitator in knowledge building within the specialty through an international collaborative network, as well as in the standardization of diagnostic processes, with translations and cross-cultural adaptations tailored to the characteristics of each population.

The COIL methodology has been applied in various health-related fields, although it is still in its early stages in the field of Speech-Language Pathology, fostering the acquisition of transversal skills such as critical thinking, self-confidence, cultural competence, teamwork, and foreign language proficiency, while simultaneously contributing to the refinement of clinical skills^(6)^. Likewise, COIL projects based on cognitive awareness laboratories stand out for their positive effects, international understanding, and the development of interpersonal skills^(7)^. Its implementation has been associated with an enriching academic experience, reflected in high levels of satisfaction and an expanded understanding of new healthcare systems^(8)^. However, it is important to highlight that, to date, no published studies have explored the use of the COIL methodology in the field of OM, which limits the analysis of successful experiences.

Furthermore, its flexibility allows for integration with different disciplines^(9)^, encouraging the exchange of knowledge among areas such as orthodontics and sleep medicine, among others. It also facilitates immersion in the public health policies of each country, revealing how OM is articulated within healthcare systems, levels of care, and professional training. This exchange enables students and faculty to identify opportunities for innovation in the incorporation of OM into neonatal, school, or rehabilitation programs, fostering an understanding that each population requires different approaches according to its specific needs, while respecting personal and cultural particularities.

Another relevant factor is the opportunity for students to experience different academic realities during their training, which broadens their understanding of diverse clinical and cultural contexts. This contributes to a more globalized education aligned with the demands of the current market. In the field of OM, the COIL approach also enables the comparison of validated clinical protocols, such as orofacial myofunctional assessment scales and tests for the analysis of the lingual frenulum, among other instruments. Furthermore, it promotes critical analysis of their strengths and limitations when applied in contexts with different languages, public health structures, and orofacial morphological characteristics specific to each population. It also encourages the pursuit and application of internationally agreed-upon knowledge regarding diagnostic criteria and therapeutic strategies for oromotor disorders, such as temporomandibular disorders, facial paralysis, or orofacial dysfunctions related to the aging process, among others, thus fostering a more uniform, ethical, and contextually grounded approach in the education of future professionals.

The results of a scoping review published in 2025, which analyzed the literature from the past ten years on the implementation, effectiveness, and challenges of COIL in undergraduate education within health professions, reinforce that this methodology promotes the development of cultural competence, communication skills, and global health awareness among students^(10)^. This review represents the first study to specifically address this topic. However, it presents methodological limitations, as being a scoping review, it does not allow for inferences about the effectiveness of the COIL methodology but rather provides an overview of the studies published to date.

Another study, published in 2022, aimed to document the experiences of faculty members at a Mexican university and identify the technological skills and intercultural competencies that, from their perspective, students develop when participating in a COIL course. The study identified three key elements for its successful implementation: training and institutional incentives, effective use of information and communication technologies, and proficiency in a foreign language^(11)^. Its findings corroborate the results of the previous review, as well as those of several other studies published on the topic.

However, its implementation requires overcoming several challenges, as emphasized by previous studies, including language barriers, the need for curricular adaptation, time zone differences, and faculty training for the use of active methodologies in distance education^(10)^. The adoption of COIL in Speech-Language Pathology, particularly in OM, demands institutional and faculty commitment to implementing academic policies that promote international collaboration as a fundamental component of higher education training.

To ensure the effective implementation of COIL in OM, it is essential to address the identified challenges through concrete strategies. Faculty training may include workshops on teaching methodologies in virtual environments and the development of intercultural competencies. Curricular adaptation, in turn, should consider the structured integration of COIL into academic programs, ensuring that the content is complementary among participating institutions. Moreover, the use of accessible teaching platforms is key to minimizing technological inequalities, prioritizing tools that enable both synchronous and asynchronous interaction, facilitate the management of time zone differences, and provide automatic translation and accessibility for students with diverse communicative and linguistic needs.

In this context, given the growing need for internationalization in Speech-Language Pathology, the implementation of COIL in OM training represents a strategic opportunity for professional qualification and the expansion of global scientific production. We invite institutions, researchers, and educators not only to explore this methodology but also to establish interinstitutional collaborations that enable its practical application.

In this regard, the creation of specialized academic networks in OM through COIL projects would significantly strengthen the clinical, theoretical, and ethical training of future professionals, while also fostering a research-oriented and collaborative attitude. The joint production of educational materials and validation proposals can contribute to the consolidation of a scientific body of knowledge specific to OM, while allowing for the recognition and respect of the cultural, morphofunctional, and healthcare model particularities of each country.
