# Supplementary material for: The relevance of the Collaborative Online International Learning methodology in global training in Orofacial Motricity
Source: Codas. 2026 Jan 26;38(1):e20250117. doi: 10.1590/2317-1782/e20250117en (PMC12978318; doi:10.1590/2317-1782/e20250117en)
Supplement: Translation

# Translation: La relevancia de la metodología *Collaborative Online International Learning* en la formación global en Motricidad Orofacial

**DOI:** 10.1590/2317-1782/e20250117es

**Authors:** Lucas Ferreira, Felipe Henríquez, Paola Leguízamo, Maria Luiza Lopes Timóteo de Lima, Hilton Justino da Silva

**Affiliations:** 1 Programa de Pós-graduação em Saúde da Comunicação Humana – PPGSCH, Universidade Federal de Pernambuco – UFPE - Recife (PE), Brasil.; 2 Departamento de Salud, Universidad de Los Lagos - Osorno, Chile.; 3 Programa de Fonoaudiología, Escuela Colombiana de Rehabilitación – ECR – Bogotá D.C., Colombia.; 4 Programa de Pós-graduação em Saúde da Comunicação Humana, Departamento de Fonoaudiologia, Universidade Federal de Pernambuco – UFPE - Recife (PE), Brasil.

## Estimadas Editoras Jefas de la Revista CoDAS,

La internacionalización de la enseñanza y la investigación en Fonoaudiología se ha consolidado como una estrategia esencial para el avance del área, especialmente en el campo de la Motricidad Orofacial (MO), comprendida como el campo de la Fonoaudiología dedicada al estudio, investigación, prevención, evaluación, diagnóstico, desarrollo, habilitación, perfeccionamiento y rehabilitación de los aspectos estructurales y funcionales de las regiones orofacial y cervical^(1)^.

El acceso a diferentes enfoques teóricos, metodológicos y clínicos amplía las posibilidades de formación e innovación. En este contexto, la metodología *Collaborative Online International Learning* (COIL) surge como una herramienta pedagógica capaz de conectar a estudiantes, docentes e investigadores de todo el mundo, promoviendo un entorno de aprendizaje colaborativo e intercultural^(2)^. En el área de la MO, esta metodología se presenta como una estrategia innovadora y pertinente para el estudio comparado de parámetros orofaciales entre poblaciones de diferentes países, favoreciendo el análisis crítico de métodos de evaluación e intervención y permitiendo la identificación de similitudes y diferencias culturales, clínicas y epidemiológicas en relación con los trastornos orofaciales.

La metodología COIL se destaca como un enfoque innovador que utiliza tecnologías en línea para viabilizar el aprendizaje global y el intercambio simultáneo de conocimientos, culturas y formas de pensamiento en el ámbito académico^(3)^. A través de esta interacción virtual entre instituciones de diferentes países, se produce una construcción conjunta del conocimiento sin la necesidad de movilidad física. Además, el COIL integra el enfoque educativo socioconstructivista, que postula que el conocimiento científico no es un conjunto neutral de datos independientes de las prácticas culturales, sino una construcción social que emerge en el contexto de las sociedades^(4)^, enfatizando el aprendizaje a través de la interacción social, un aspecto esencial para el desarrollo de la competencia intercultural^(5)^.

Esta metodología conecta educadores de diferentes regiones para desarrollar un programa compartido en línea^(5)^. Este proceso incluye actividades virtuales grupales en las que los estudiantes colaboran para cocrear el aprendizaje. Por ejemplo, un profesor de una universidad A puede establecer una colaboración con un profesor de una universidad internacional B para crear un curso o una clase conjunta, en donde además se privilegia el intercambio cultural. En este contexto, dos instituciones, dos profesores y dos grupos de estudiantes participan activamente, lo cual puede involucrar más de un idioma, diferentes perspectivas y diferentes tópicos académicos, de manera colaborativa, con tareas desarrolladas en un entorno virtual, promoviendo la cooperación entre los estudiantes y la interdisciplinariedad.

A través de este tipo de proyectos colaborativos, los estudiantes pueden investigar diversas aristas de la MO, como, por ejemplo, la respiración oral, los trastornos de masticación, la deglución disfuncional, los trastornos del sueño o las alteraciones del habla de origen musculoesquelético entre otros temas. Esta experiencia estimula la discusión sobre el desarrollo de métodos de evaluación válidos y herramientas clínicas adaptadas a cada realidad, promoviendo una práctica basada en la equidad y la diversidad.

De este modo, la búsqueda de una MO globalizada puede lograrse cuando la enseñanza y la producción de conocimiento entre diferentes instituciones de educación superior siguen un enfoque más alineado, reduciendo discrepancias en relación con la nomenclatura, los procesos de evaluación y las estrategias terapéuticas de los trastornos miofuncionales orofaciales. Esta metodología puede actuar como un facilitador en la construcción del conocimiento dentro de la especialidad, a través de una red colaborativa internacional, así como en la estandarización de los procesos diagnósticos, con traducciones y adaptaciones transculturales que se adecuen a las características de cada población.

La metodología COIL ha sido utilizada en diversas áreas de la salud, aunque de manera incipiente en la Fonoaudiología, favoreciendo la adquisición de habilidades transversales como el pensamiento crítico, la autoconfianza, la competencia cultural, el trabajo en equipo y la competencia en lengua extranjera; al mismo tiempo que contribuye al perfeccionamiento de las habilidades clínicas^(6)^. De igual manera, los COIL basados en laboratorios de conciencia cognitiva destacan por sus efectos positivos, comprensión internacional y desarrollo de habilidades interpersonales^(7)^. Su aplicación ha sido asociada con una experiencia académica enriquecedora, reflejada en altos niveles de satisfacción y en una comprensión ampliada de nuevos sistemas de salud^(8)^. No obstante, es importante destacar que, hasta el momento, no existen estudios publicados sobre el uso de la metodología COIL en el área de la MO, lo que limita el análisis de experiencias exitosas.

Por otra parte, se destaca que su flexibilidad permite la integración con diferentes disciplinas^(9)^, incentivando el intercambio de conocimientos entre áreas como ortodoncia y la medicina del sueño entre otras, además, facilita una inmersión en las políticas públicas de salud de cada país, revelando cómo la MO se articula en los sistemas de salud, los niveles de atención y la formación profesional. Este intercambio permite a estudiantes y docentes identificar oportunidades de innovación en la inserción de la MO en programas de atención neonatal, escolar o en contextos de rehabilitación, comprendiendo como cada población requiere de diferentes enfoques de acuerdo con sus necesidades, respetando las particularidades personales y culturales.

Otro factor relevante es la posibilidad de que el estudiante experimente diferentes realidades académicas durante su formación, lo que amplía su percepción sobre contextos clínicos y culturales diversos. Esto contribuye a una formación más globalizada, alineada con las demandas del mercado actual. En el ámbito de la MO, el enfoque COIL también permite la comparación de protocolos clínicos validados, como escalas de evaluación miofuncional orofacial y pruebas para el análisis del frenillo lingual, entre otros instrumentos. Además, fomenta el análisis crítico de sus fortalezas y limitaciones al ser aplicados en contextos con distintos idiomas, estructuras de salud pública y características morfológicas orofaciales propias de cada población. Asimismo, estimula la búsqueda y el uso de conocimientos consensuados internacionalmente sobre los criterios diagnósticos y las estrategias terapéuticas para trastornos oromotores, como los desórdenes temporomandibulares, la parálisis facial o las disfunciones orofaciales vinculadas al proceso de envejecimiento, entre otras, lo que favorece una aproximación más uniforme, ética y contextualizada en la formación de los futuros profesionales.

Los resultados de un *scoping review* publicada en 2025, que analizó la literatura disponible en los últimos 10 años sobre la implementación, efectividad y desafíos del COIL en la formación de pregrado en las profesiones de la salud, refuerza que esta metodología favorece el desarrollo de la competencia cultural, de las habilidades comunicativas y de la conciencia sobre la salud global entre los estudiantes^(10)^. Dicha revisión constituye el primer estudio que abordó específicamente este tema. No obstante, presenta limitaciones metodológicas, ya que, al tratarse de una *scoping review*, no permite inferir la efectividad de la metodología COIL, sino que proporciona una visión general de los estudios publicados.

Otro estudio, publicado en 2022, que buscó recuperar las experiencias de los académicos de una universidad mexicana e identificar las habilidades tecnológicas y competencias interculturales que, desde su perspectiva, desarrollan sus estudiantes al participar en un curso COIL, identificó tres elementos clave para su implementación exitosa: la capacitación y los incentivos institucionales, el aprovechamiento de las tecnologías de la información y la comunicación, y el dominio de otro idioma^(11)^. Sus hallazgos corroboran los resultados de la revisión anterior, así como de diversos estudios publicados sobre el tema.

Sin embargo, su implementación requiere superar desafíos, como los enfatizados por los estudios anteriores, y relacionados a las barreras lingüísticas, la necesidad de adecuación curricular, las diferencias horarias y la capacitación docente para el uso de metodologías activas en la enseñanza a distancia^(10)^. La adopción del COIL en la Fonoaudiología, particularmente en la MO, exige el compromiso institucional y docente, en la implementación de políticas académicas que fomenten la colaboración internacional como parte fundamental de la formación superior.

Para garantizar una implementación efectiva del COIL en la MO, es fundamental abordar los desafíos identificados mediante estrategias concretas. La capacitación docente puede incluir talleres sobre metodologías de enseñanza en entornos virtuales y el desarrollo de competencias interculturales. La adaptación curricular, por su parte, debe considerar la integración del COIL en los planes de estudio de manera estructurada, asegurando que los contenidos sean complementarios entre las instituciones participantes. Además, el uso de plataformas de enseñanza accesibles es clave para minimizar desigualdades tecnológicas, priorizando herramientas que permitan la interacción sincrónica y asincrónica, facilitando la gestión de diferencias horarias, así como, la traducción automática y la accesibilidad para estudiantes con diferentes necesidades comunicativas y lingüísticas.

Así, dada la creciente necesidad de internacionalización en la Fonoaudiología, la implementación del COIL en la formación en MO representa una oportunidad estratégica para la cualificación profesional y la expansión de la producción científica global. Invitamos a instituciones, investigadores y docentes a no solo explorar está metodología, sino también, a establecer colaboraciones interinstitucionales que permitan su aplicación concreta.

En este sentido, la creación de redes académicas especializadas en MO mediante proyectos COIL, fortalecería de forma significativa la formación clínica, teórica y ética de los futuros profesionales, además de fomentar una actitud investigativa y colaborativa. La producción conjunta de materiales educativos y propuestas de validación puede contribuir a la consolidación de un cuerpo de conocimientos científicos propios de la MO, al tiempo que permite reconocer y respetar las particularidades culturales, morfofuncionales y de los modelos de atención en salud de cada país.
